# What is behind the chest pain? Rare esophageal hematoma caused by betel nut: A case report

**DOI:** 10.1002/deo2.70074

**Published:** 2025-02-10

**Authors:** Chundi Guan, Meng Yang Wang, Xingfang Jia

**Affiliations:** ^1^ Department of Gastroenterology Binzhou Medical University Hospital Binzhou China; ^2^ Department of Gastroenterology Qilu Hospital of Shandong University Jinan China

**Keywords:** areca, esophagus, gastroscopes, hematoma, ulcer

## Abstract

This case report reported a case of huge esophageal hematoma caused by chewing betel nut. The patient was admitted to the hospital due to chest pain. After a multidisciplinary diagnosis by the Department of Gastroenterology, Cardiology, and Radiology, it was finally determined that the patient's chest pain was caused by a huge esophageal hematoma. After diagnosis and treatment, we observed the longitudinal ulcer after the healing of the esophageal giant hematoma. This case report provides evidence that chewing betel nuts may be a cause of gastrointestinal bleeding.

## INTRODUCTION

Betel nut is a palm tree evergreen tall tree, native to the tropical rainforests of the Malay Peninsula. It is now widely distributed in India, Pakistan, Sri Lanka, Malaysia, New Guinea, Indonesia, the Philippines, Myanmar, Thailand, Vietnam, and Cambodia. Because betel nut has the functions of removing dampness and cold, stimulating the mind and spirit, promoting digestion, and addiction, some people prefer it. However, chewing betel nut has many adverse effects, and we have found that it also has the risk of gastrointestinal bleeding. This article introduces a case of esophageal hematoma caused by chewing betel nut. Therefore, in addition to preventing oral cancer, we should also be vigilant against the possibility of gastrointestinal bleeding caused by chewing betel nut.

## CASE REPORT

**FIGURE 1:**
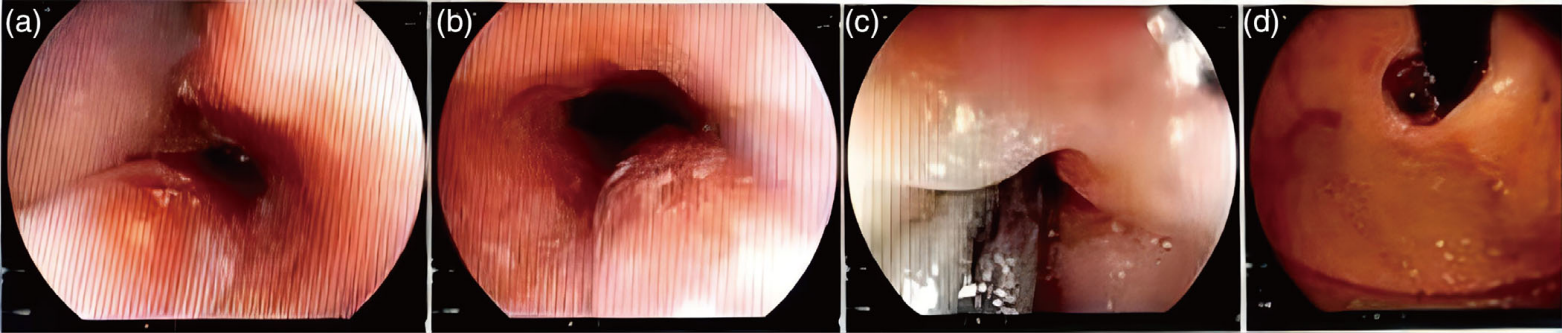
Esophageal hematoma of the esophagogastroduodenoscopy sight. From left to right, they are as follows. (a) A cord‐like bluish‐purple eminence was observed 25 cm from the incisor. (b) Another perspective for observing the bulge after rotating endoscopy. (c) Push the endoscope to observe the bulge at a close point, it can be seen that the top of the proximal end of the bulge is an erosion area, and the texture of the touch is soft. (d) After entering the stomach, the reverse endoscope showed that the bulge extended to the right posterior wall of the cardia.

A 62‐year‐old man who had suffered pain under the xiphoid process for 4 days was admitted to the ward on a stretcher. Besides the abdominal pain, tarry stool is another severe symptom which suggests that this patient may have experienced gastrointestinal bleeding. Without any remarkable physical examination, this patient reported no other discomfort or obvious symptoms. An esophagogastroduodenoscopy report was obtained from the local endoscopy center, which diagnosed the esophageal mucosa 25–30 cm away from the incisor was hyperemic and edematous, and the lumen was obviously narrow, no erosion or ulcer was observed, and the mucosa below was blue‐purple in color, extending into the stomach cavity. In order to prevent further mucosal injury, no further biopsy was taken (Figure 1).

He remembered that chewing betel nuts 4 days before was the cause of the abdominal pain, and had no long‐term history of chewing betel nuts, this was the first time that betel nut was taken. Moreover, this patient denied any other trauma or medical history. We excluded the differential diagnosis of causes including esophageal foreign body injury, rapid changes in in vitro pressure, and coagulation dysfunction.

**FIGURE 2:**
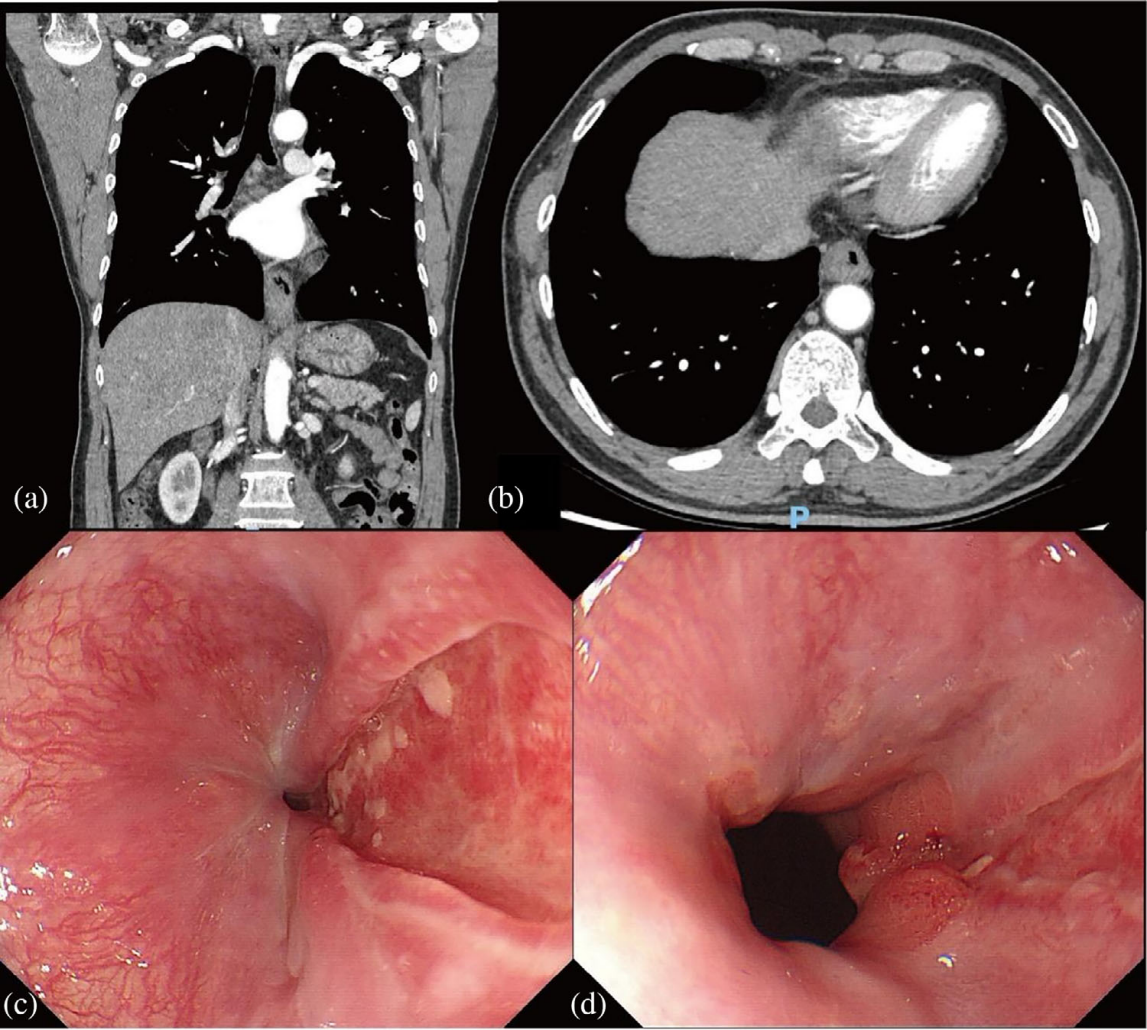
Reexamination of esophageal hematoma after treatment. (a, b) A computed tomography scan showed the wall of the esophagus gastric fundus and cardia were thickened. (c, d) The second esophagogastroduodenoscopy showed longitudinal ulcers.

After the patient was admitted to the hospital, there were no obvious abnormalities in the patient's blood routine, biochemical, coagulation function, or other test results. The cardiologist first performed electrocardiogram and echocardiography. After ruling out cardiac disease, the radiologist conducted a consultation on the computed tomography images of the patient and pointed out that lower esophageal edema was more obvious. During the period of hospitalization, the patient was prohibited from diet temporarily. We gave the patient a parenteral nutrition regimen based on the consultation opinion of the nutrition department. Moreover, Esomeprazole and levofloxacin were administered intravenously to inhibit gastric acid and prevent infection respectively. Sucralfate suspension gel was given orally to promote mucosal repair. Five days later, his pain and tarry stool were obviously relieved than before. Computed tomography revealed that the walls of the middle and lower segments of the esophagus and the gastric fundus and cardia were thickened (Figure 2a,b). Repeat endoscopy revealed that the edema of the duct mucosa significantly subsided. Strip ulcers appeared 30 cm away from the incisor in the original edema area, extending to the gastric fundus near the gate. The ulcers were coated with thin white fur without bleeding, and the surrounding mucosa was hyperemic. No obvious abnormality was found in the remaining gastric cavity and duodenum.

## DISCUSSION

Betel nut is a widely distributed palm plant seed in tropical and subtropical regions, mainly cultivated in the three provinces of Hainan, Taiwan, and Yunnan in China. Owing to its addictive properties, it has formed regional chewing habits. Areca nut contains various active ingredients, including alkaloids, flavonoids, tannins, triterpenes, steroids, polysaccharides, fatty acids, and amino acids. The content of biological alkaloids in the areca nut fruit is approximately 0.3%–0.6%, which is the main pharmacologically active component. Among them, the content of high areca alkaloids is between 0.1% and 0.5%, followed by arecaine, demethylcatechol, norarecaine, demethylnorarecaine, etc.^1^ The betel nut has certain pharmacological effects on the digestive system, nervous system, anti‐depression, cardiovascular system, anti‐inflammation, and anti‐virus.^2^

Betel nut besides there are more than a variety of benefits, also exist many adverse reactions and hazards. Habitual use of betel nuts causes local trauma and mucosal injury. Repetitive damage leads to chronic inflammation, oral submucosal fibrosis, and oral cancer.^3^ The International Agency for Research on Cancer of the World Health Organization classified the betel nut fruit as a Group 1 carcinogen in 2004.^4^ There is evidence that areca alkaloids further promote oral submucosal fibrosis by inducing transforming growth factor beta signal activation in oral epithelial cells.^5^, ^6^

Interestingly, digestive tract bleeding and esophageal hematoma caused by betel nuts were reported as rare cases of betel nuts‐related adverse events in recent years. In 2022, Gao reported two cases of severe esophageal injury caused by chewing betel nut.^7^ Wang reported one case of esophageal hematoma caused by chewing betel nut.^8^ This patient in this study also presented a rare case of esophageal hematoma caused by chewing betel nut. These two reports also dealt with esophageal injury caused by betel nut but did not specifically mention such severe hematoma formation. This report not only describes the formation of the hematoma but also records the treatment process and the healing process of the large ulcer after the hematoma. By means of gastroscopy and computed tomography scanning, the complete process from hematoma to ulcer to healing was demonstrated, which has a high reference value in the clinic.

In addition, some studies have compared the effects of three different consuming methods of fresh betel nut on mouse immune function. The results revealed that the consumption method of smoke‐dried tobacco leaf is the most obvious in reducing mouse immune function, followed by the consumption method of betel nut leaves and that the consumption method of fresh betel nut has a relatively small effect.^9^ The three consumption methods of fresh betel nut have different degrees of negative effects on mouse weight and immune function, and the consumption methods of smoke‐dried tobacco leaf and betel nut leaves have a more obvious negative effect on mouse immune function. Therefore, it is particularly important to process and regulate betel nut in a proper way, paying attention to strengthening its management and using it reasonably while minimizing its harm to human bodies. We are still unable to identify the exact mechanism of esophageal hematoma in this case, and further basic experiments are needed to further explore the cause of esophageal hematoma. This exploration of the underlying mechanism provides clues and directions for future research. Secondly, reducing quicklime, tobacco, and other ingredients often added in the processing of betel nut may reduce the synergic damage caused by digestive tract mucosa. This case report reported for the first time a case of esophageal hematoma caused by chewing betel nut in northern China, and the treatment of this case involved multiple disciplines such as gastroenterology, imaging, and cardiology, emphasizing the importance of multidisciplinary cooperation in the treatment of hematoma caused by chewing betel nut. In addition, the treatment of this case involved gastroenterology, imaging, cardiology, and other multidisciplinary disciplines, emphasizing the importance of multidisciplinary cooperative treatment of hematoma caused by chewing areca nut, and providing a reference method for diagnosis and treatment in areas where chewing areca nut is becoming increasingly popular.

## CONFLICT OF INTEREST STATEMENT

None.

## ETHICS STATEMENT

The image authorization and publication permission were obtained from the Digestive Endoscopy Center of the Affiliated Hospital of Binzhou Medical College.

## PATIENT CONSENT STATEMENT

The written consent of the patient has been obtained.

## CLINICAL TRIAL REGISTRATION

N/A.
